# Prevalence of heterozygous and homozygous 9p21 deletions in human cancer: a tissue microarray study on 4,999 tumors from 125 different tumor types

**DOI:** 10.1186/s10020-026-01542-0

**Published:** 2026-06-27

**Authors:** Natalia Gorbokon, Katharina Teljuk, Nina Schraps, Maximilian Lennartz, Sebastian Dwertmann Rico, Simon Kind, Viktor Reiswich, Florian Viehweger, Florian Lutz, Christoph Fraune, Andreas M Luebke, Anne Menz, Ria Schlichter, Till Krech, Andrea Hinsch, Eike Burandt, Guido Sauter, Ronald Simon, Stefan Steurer, Andreas H Marx, Patrick Lebok, David Dum, Sarah Minner, Frank Jacobsen, Till S Clauditz, Thilo Hackert, Faik G. Uzunoglu, Christian Bernreuther, Martina Kluth

**Affiliations:** 1 Institute of Pathology, University Medical Center Hamburg-Eppendorf, Martinistrasse 52, Hamburg, 20246 Germany; 2 Institute of Pathology, Clinical Center Osnabrueck, Osnabrueck, Germany; 3 Department of Pathology, Academic Hospital Fuerth, Fuerth, Germany; 4 Department of General, Visceral and Thoracic Surgery, University Medical Center Hamburg-Eppendorf, Hamburg, Germany

**Keywords:** MTAP deficiency, 9p21 deletion, FISH, IHC, Tissue microarray, human cancers

## Abstract

**Background:**

Homozygous 9p21 deletions are the major cause for MTAP deficiency, making cancer cells more vulnerable towards drugs targeting various pathways. This study aimed to assess the prevalence of heterozygous and homozygous 9p21 deletions in cancer.

**Methods:**

A tissue microarray containing 7,172 samples from 125 different tumor entities was analyzed by fluorescence in situ hybridization. This method allows the detection of deletions within single tumor cells, while admixted non-neoplastic cells are omitted. Consecutive sections were immunostained for MTAP and p16 (*CDKN2A*).

**Results:**

Among 4,999 evaluable tumors, 10.7% had heterozygous and 7.4% homozygous deletions, which occurred in different tumor categories. Homozygous deletions were most frequent in mesothelioma (up to 47.8%), pancreatic ductal adenocarcinoma (44.7%), and urothelial carcinoma (up to 36.4%) while heterozygous deletions predominated in squamous cell carcinoma (up to 34.3%), leiomyosarcoma (31.4%), and esophageal adenocarcinoma (30.8%). The proportion of homozygous 9p21 deletions was high in mesothelioma (up to 91.6%), urothelial carcinoma (up to 80.0%), and pancreatic ductal adenocarcinoma (76.4%), intermediate in squamous cell carcinomas of different organs (12.5–50.0%) and pulmonary adenocarcinoma (46.3%), and low in endometrioid (9.1%) and high-grade serous (4.0%) ovarian carcinoma. MTAP and p16 immunostaining was absent in homozygous 9p21 deletions, while heterozygously deleted cancers showed markedly decreased MTAP immunostaining (*p* < 0.0001), with unchanged p16 staining.

**Conclusions:**

These data provide a comprehensive overview on the prevalence on homozygous and heterozygous 9p21 deletions in cancer and demonstrate that different cancer types markedly differ in their ratio of homozygous/heterozygous 9p21 deletions. The strong concordance between homozygous 9p21 deletions and absent MTAP immunostaining highlights the effectiveness of immunohistochemistry in detecting MTAP deficiency.

**Supplementary Information:**

The online version contains supplementary material available at 10.1186/s10020-026-01542-0.

## Introduction

9p21 deletions belong to the most common chromosomal aberrations in cancer. Its most known target gene is *CDKN2A* - a cell cycle regulator gene - coding for the p16 protein which plays a critical role in many tumors (summarized in (Zhao et al. 2016)). However, most of the topical interest in 9p21 comes from S-methyl-5′-thioadenosine phosphorylase (*MTAP*) gene which is also included in most 9p21 deletions. The *MTAP* gene is located at 9p21.3, only 30 kb apart from the *CDKN2A* gene which is thought to be homozygously deleted in up to 15% of cancers (Beroukhim et al. 2010, Consortium 2020, Harrison et al. 2023). MTAP is essential for the salvage pathway of adenine synthesis (Della Ragione et al. 1986) and MTAP deficiency results in a critical vulnerability of cancer cells towards drugs targeting different pathways (Alhalabi et al. 2022). As adenine synthesis can only be maintained by de novo biosynthesis in MTAP deficient cancer cells, inhibition of critical enzymes for folate synthesis leads to the death of MTAP deficient cancer cells in experimental models and also showed anti-cancer efficiency in urinary bladder cancer patients (Alhalabi et al. 2022). MTAP deficient cells can also be successfully targeted by inhibitors of protein arginine N-methyltransferase 5 (PRMT5) and methionine adenosyltransferase II alpha (MAT2A) (summarized in (Bray et al. 2023)). PRMT5 is essential for the methylation of numerous proteins thus regulating their activity (Blanc and Richard 2017). MAT2A is critically needed for the synthesis of S-adenosylmethionine (SAM), the methyl donor and substrate of PRMT5 (Lu and Mato 2012, Murray et al. 2016). As PRMT5 efficiency is already hindered in MTAP deficient tumor cells by the accumulation of the unprocessed MTAP metabolite MTA (Kryukov et al. 2016), these cells are more susceptible to further PRMT5 suppression by PRMT5 or MAT2A inhibiting drugs (summarized in (Bray et al. 2023)). Recently, a significant reduction of tumor size was reported after treatment of patients with different types of MTAP deficient cancer types with the PRMT5 inhibitor MRTX1719 (Engstrom et al. 2023). Monotherapy by the MAT2A inhibitor IDE397 resulted in one complete and 8 partial remissions (PR) in a cohort of 27 MTAP deficient lung and bladder cancer patients which had received several lines of prior chemotherapy (Herzberg and Johnson 2024).

Immunohistochemistry (IHC) using specific MTAP antibodies has proven to be an excellent surrogate marker for homozygous *MTAP* deletions at 9p21 because the protein is ubiquitously expressed at high levels and MTAP staining is completely lost in cells lacking both *MTAP* gene copies (Gorbokon et al. 2024). In an earlier study on 13,067 tumors, we had identified MTAP deficiency by immunohistochemistry (IHC) in 83 of 149 different tumor entities (Gorbokon et al. 2024). FISH validation including all cases with MTAP expression loss and all tumors from three selected tumor types had revealed that with the exception of neuroendocrine neoplasms and lymphomas, MTAP deficiency was regularly caused by homozygous MTAP deletion. Since heterozygous 9p21 deletion is likely to precede homozygous deletion, we were interested in the prevalence of heterozygous 9p21 (*MTAP)* deletions in different type of cancers. However, data on heterozygous 9p21 deletions are highly variable in the literature. For example, the rate of heterozygous 9p21 deletions range from 11.0 to 36.0% in mesothelioma (Ito et al. 2015, Onofre et al. 2008) and from 3.0 to 77.0% in bladder cancer (Orlow et al. 1999, Bartoletti et al. 2007).

Fluorescence in situ hybridization (FISH) is the gold standard method for detection of large chromosomal gene deletions because single tumor cells can be identified and analyzed independent of admixed non-neoplastic cells (Puiggros et al. 2014, Lotan et al. 2016). To comprehensively determine the prevalence of heterozygous and homozygous *MTAP* deletions in different tumor entities, a tissue microarray (TMA) containing 7,172 samples from 125 different tumor entities was analyzed by FISH in this study and the results were compared with p16 and MTAP IHC data obtained on consecutive tissue section.

## Materials and methods

### Tissue microarrays (TMAs)

Our TMA was composed of one sample measuring 0.6 mm each from 7,172 primary tumors from 125 different tumor types and subtypes as previously described (Gorbokon et al. 2024). The composition of the TMAs is described in detail in the results section. All TMAs were analyzed by FISH and IHC on consecutive TMA slides. All samples were from the archives of the Institute of Pathology, University Hospital of Hamburg, Germany, the Institute of Pathology, Clinical Center Osnabrueck, Germany, and the Department of Pathology, Academic Hospital Fuerth, Germany. Tissues were fixed in 4% buffered formalin and then embedded in paraffin. The use of archived remnants of diagnostic tissues for TMA manufacturing, their analysis for research purposes, and the use of patient data were according to local laws (HmbKHG, § 12) and analysis had been approved by the local ethics committee (Ethics commission Hamburg, WF-049/09). All work has been carried out in compliance with the Helsinki Declaration.

### Fluorescence in situ hybridization (FISH)

Five micrometer TMA sections were deparaffinized with xylol, rehydrated through a graded alcohol series and exposed to heat-induced denaturation for 10 minutes in a water bath at 99°C in P1 pretreatment solution (Agilent Technologies, Santa Clara, CA, USA; #K5799). For proteolytic treatment, slides were added to VP2000 protease buffer (Abbott, Chicago, IL, USA; #2J.0730) for 200 minutes at 37°C in a water bath. A commercial FISH probe kit containing both, a 9p21 probe including both the *CDKN2A* and the *MTAP* gene and a centromere 9 probe were utilized for 9p21 copy number detection (ZytoLight ^®^ SPEC CDKN2A/CEN 9 Dual Color Probe, Zytovision, Bremerhaven, Germany; #Z-2063). Hybridization was performed overnight at 37°C in a humidified chamber. Posthybridization washes were done according to the manufacturer’s direction at 37°C (Agilent Technologies, Santa Clara, CA, USA; #K5799). Nuclei were counterstained with 125 ng/ml 4’,6-diamino-2-phenylindole in antifade solution (Biozol; Eching, Germany; #VEC-H-1200). Stained tissues were manually interpreted with an epifluorescence microscope and copy numbers of 9p21 and centromere 9 were estimated for each tissue spot. Presence of equal numbers of 9p21 and centromere 9 signals in tumor cell nuclei was considered as 9p21 normal. Presence of fewer 9p21 signals than centromere 9 probe signals in at least 60% tumor cell nuclei or one 9p21 and one centromere 9 signal (monosomy of chromosome 9) in nearly all tumor cell nuclei of a tissue spot (at least 25 cell nuclei to more than 1,000) were considered as heterozygous deletion. Complete absence of 9p21 signals but presence of centromere 9 signals in the tumor cell nuclei and presence of unequivocal 9p21 and centromere 9 signals in tumor adjacent normal cell nuclei, was considered homozygous 9p21 deletion. This threshold was based on a previous validation study comparing *PTEN* copy number results analyzed by FISH and array comparative genomic hybridization in prostate cancer (Krohn et al. 2012). Tissue spots lacking any detectable 9p21 signals in all (tumor and normal cell nuclei) or lack of any normal cells as an internal control for successful hybridization of the 9p21 probe were excluded from analysis.

### Immunohistochemistry (IHC)

Freshly prepared 2.5 μm TMA sections that were consecutive to the slide used for FISH were analyzed on one day for p16 and MTAP. MTAP staining was executed in a Dako Omnis automated stainer as previously described (Agilent Technologies, Santa Clara, CA, USA) using the EnVision FLEX, High pH Kit (Agilent Technologies, Santa Clara, CA, USA, #GV800). Slides were deparaffinized with Clearify™ agent (Agilent Technologies, Santa Clara, CA, USA, #GC810) and exposed to heat-induced antigen retrieval for 30 min at 97 °C in Target Retrieval Solution, High pH reagent (part of Agilent kit #GV800). Primary antibody specific for MTAP (recombinant rabbit monoclonal, MSVA-741R, MS Validated Antibodies GmbH, Hamburg, Germany, #5293-741R) was applied at ambient temperature for 30 min at a dilution of 1:50. Endogenous peroxidase activity was blocked with Peroxidase-Blocking-Reagent (part of Agilent kit #GV800) for 3 min. Bound antibody was visualized using the EnVision FLEX, High ph kit reagents DAB+ Chromogen and Substrate Buffer (parts of Agilent kit #GV800) and EnVision FLEX + Rabbit LINKER (Agilent Technologies, Santa Clara, CA, USA; #GV809) according to the manufacturer’s directions. The sections were counterstained with hemalaun. For tumor tissues, the average MTAP staining intensity of all unequivocally neoplastic cells was estimated as 0, 1+, 2+, 3 + as previously described (Gorbokon et al. 2024, Gorbokon et al. 2025). For the classification of a tumor as completely negative (0), presence of unequivocal MTAP staining in tumor adjacent normal tissue was required. Tumors with complete absence of MTAP staining in cancerous cells and a lack of stromal cells with unequivocal MTAP staining were considered “non-informative”. For manual p16 staining, slides were deparaffinized with xylol, rehydrated through a graded alcohol series and exposed to heat-induced antigen retrieval for 5 min in an autoclave at 121 °C in Dako Target Retrieval Solution, pH9 (Agilent Technologies, Santa Clara, CA, USA; #S2367). Endogenous peroxidase activity was blocked with Dako REAL Peroxidase-Blocking Solution (Agilent Technologies, Santa Clara, CA, USA; #S2023) for 10 min. Primary antibody specific against p16 protein (rabbit recombinant clone MSVA-016R; MS Validated Antibodies GmbH, Hamburg, Germany) was applied at 37 °C for 60 min at a dilution of 1:150. Bound antibody was then visualized using the Dako REAL EnVision Detection System Peroxidase/DAB+, Rabbit/Mouse kit (Agilent Technologies, Santa Clara, CA, USA; #K5007) according to the manufacturer’s directions. The sections were counterstained with hemalaun. For all tumor tissues, the percentage of positive neoplastic cells was estimated, and the staining intensity was semi-quantitatively recorded (0, 1+, 2+, 3+) as previously described (Wispelaere et al. 2022). For statistical analyses, the intensity of p16 staining results were categorized into four groups (0, 1+. 2+, and 3+). Tumors without any p16 staining in all tumor cells were considered as negative.

### Statistics

Statistical calculations were performed with JMP17^®^ software (SAS^®^, Cary, NC, USA). Contingency tables and the chi²-test were performed to search for associations between 9p21 deletions and MTAP or p16 immunostaining. A likelihood ratio of ≤ 0.05 was considered as statistically significant.

## Results

### Technical issues

A total of 4,999 (69.7%) of 7,172 tumor samples were interpretable in our FISH TMA analysis. Non-interpretable samples demonstrated a lack of unequivocal tumor cells, loss of the entire tissue spot, or inefficient hybridization. Of the 4,999 FISH evaluable cancers, 4,326 (86.5%) were informative for MTAP, and 4,799 (95.9%) for p16 IHC.

### 9p21 deletion results

**Fig. 1:**
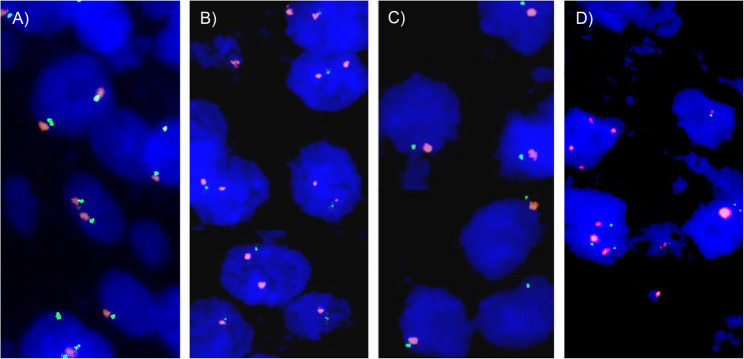
Examples of 9p21 copy number status measures by fluorescence in-situ hybridization. **A** normal 9p21 copy number status with two green 9p21 and two orange centromere 9 signals, **B** heterozygous 9p21 deletion with one green 9p21 and two orange centromere 9 signals, **C** monosomy of chromosome 9 with one green 9p21 and one orange centromere 9 signal, and **D **homozygous 9p21 deletion without any green 9p21 signal and two orange centromere 9 signals in the tumor cell nuclei and green 9p21 and orange centromere 9 signals in adjacent normal cell nuclei

**Fig. 2:**
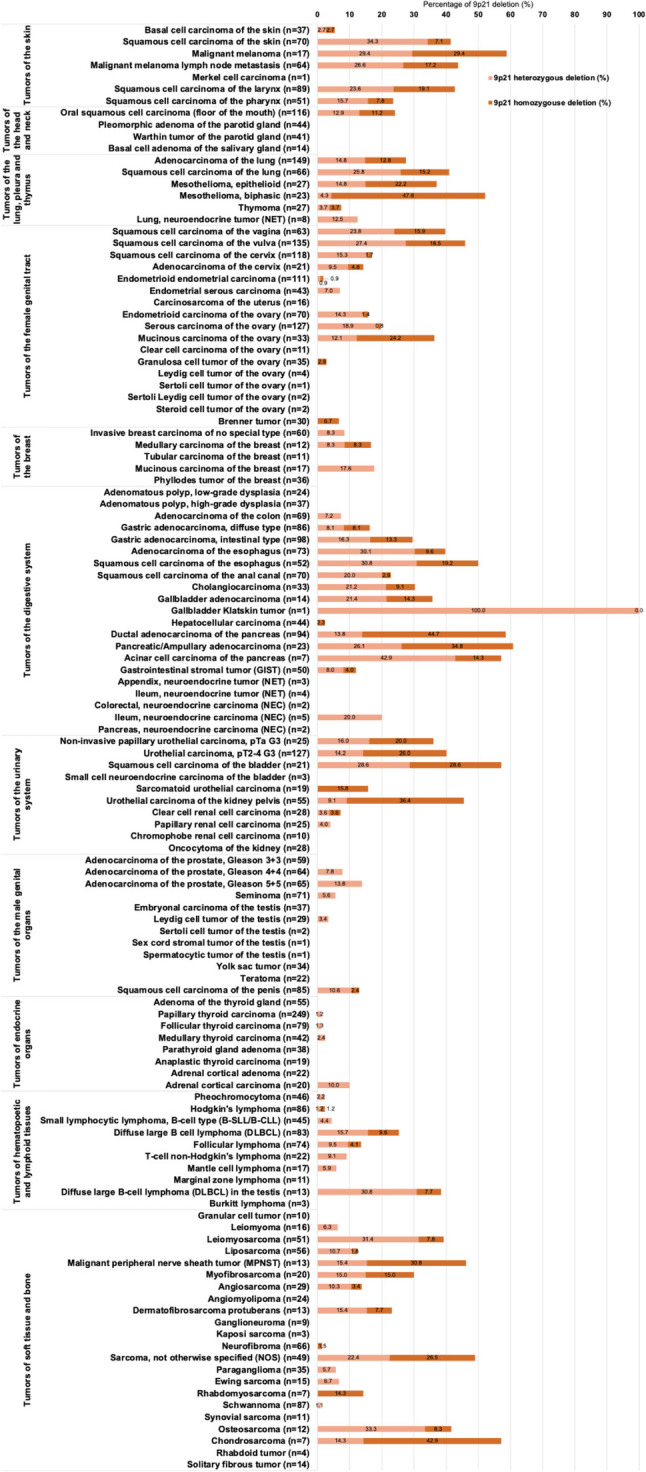
9p21 copy number status in human tumors

**Fig. 3:**
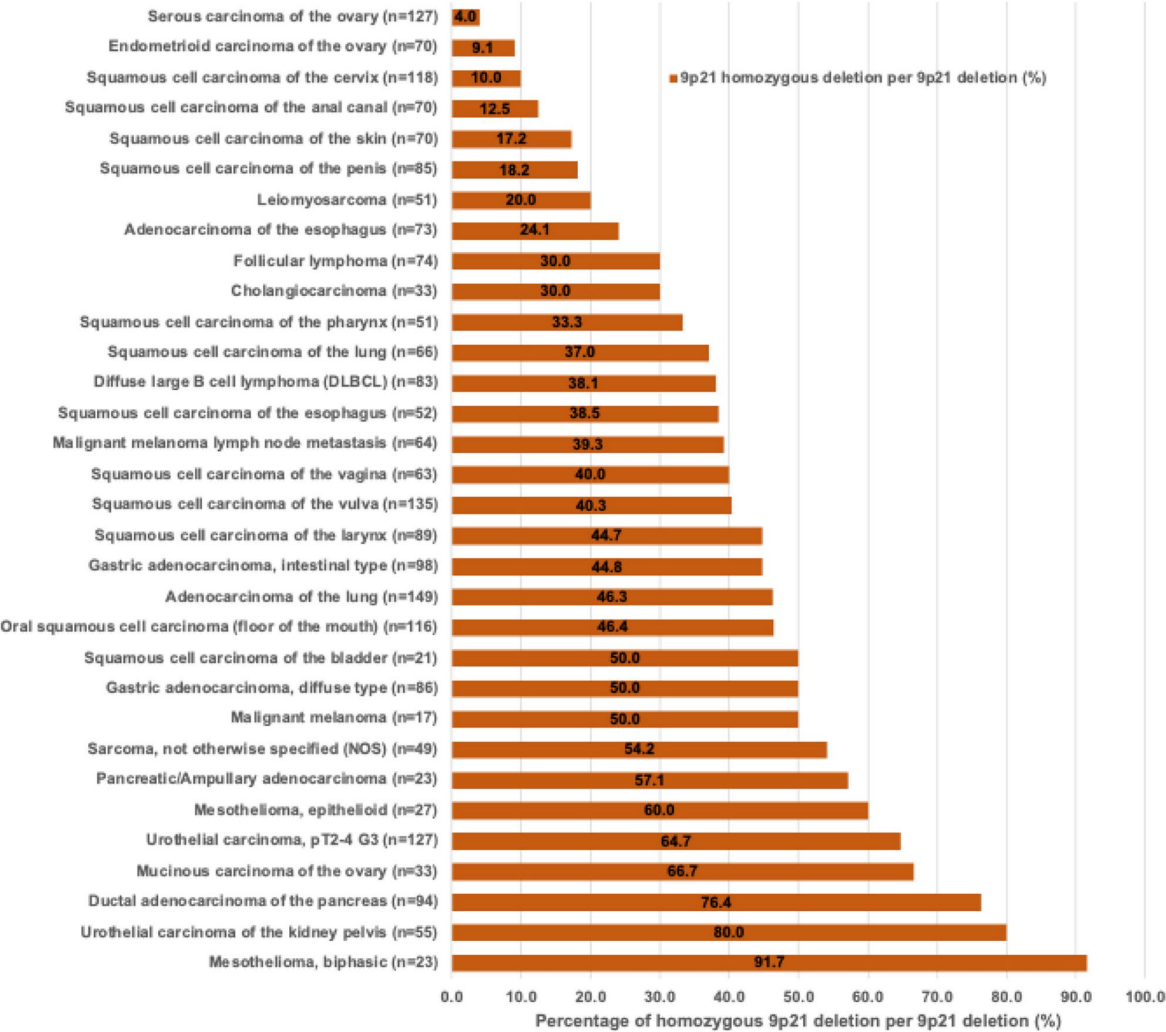
Proportion of homozygous 9p21 deletion among 9p21 deleted cancers. The figure provides a ranking order of cancers according to their ratio of homozygous 9p21 deletion per all 9p21 deletions. Tumor entities with fewer than 10 deleted cancers were excluded from this analysis

Among 4,999 evaluable tumors, 10.7% had a heterozygous, and 7.4% had a homozygous deletion. At least one case with a 9p21 deletion was found in 84 of 125 evaluable tumor entities and 58 tumor entities contained at least one tumor with a homozygous deletion. Representative images are shown in Fig. 1. For each tumor entity, the percentage of tumors with a heterozygous deletion, the percentage of tumors with a homozygous deletion, and - among the 9p21 deleted cases - the proportion of tumors with a homozygous deletion is given in Figs. 2 and 3 and Supplementary Table 1. These data show that heterozygous and homozygous 9p21 deletions do not predominate in the same tumor categories. The frequency of homozygous 9p21 deletions was highest in mesothelioma (up to 47.8%), ductal adenocarcinoma of the pancreas (44.7%), urothelial carcinoma (up to 36.4%), and in adenocarcinoma of the papilla of Vater (34.8%). In contrast, the highest rates of heterozygous 9p21 deletions occurred in squamous cell carcinoma (up to 34.3%), leiomyosarcoma (31.4%), esophageal adenocarcinomas (30.8%), malignant melanoma (29.4%), and cholangiocarcinoma (21.2%). Accordingly, the proportion of homozygous deletions within the 9p21 deleted cases were highly variable. Examples of tumors with high “relative” percentages of homozygous deletions include mesothelioma (up to 91.6% homozygous), urothelial carcinoma (up to 80.0%), pancreatic ductal adenocarcinoma (76.4%), and mucinous carcinoma of the ovary (66.7%), while squamous cell carcinomas of different sites (12.0–50.0%) and pulmonary adenocarcinoma (46.3%) represent examples of intermediate “relative” percentages of homozygous deletions. Endometrioid (9.1%) and serous carcinoma of the ovary (4.0%) constitute cancers with particularly low “relative” percentages of homozygous deletions.

### 9p21 deletion, MTAP, and p16 expression

**Fig. 4:**
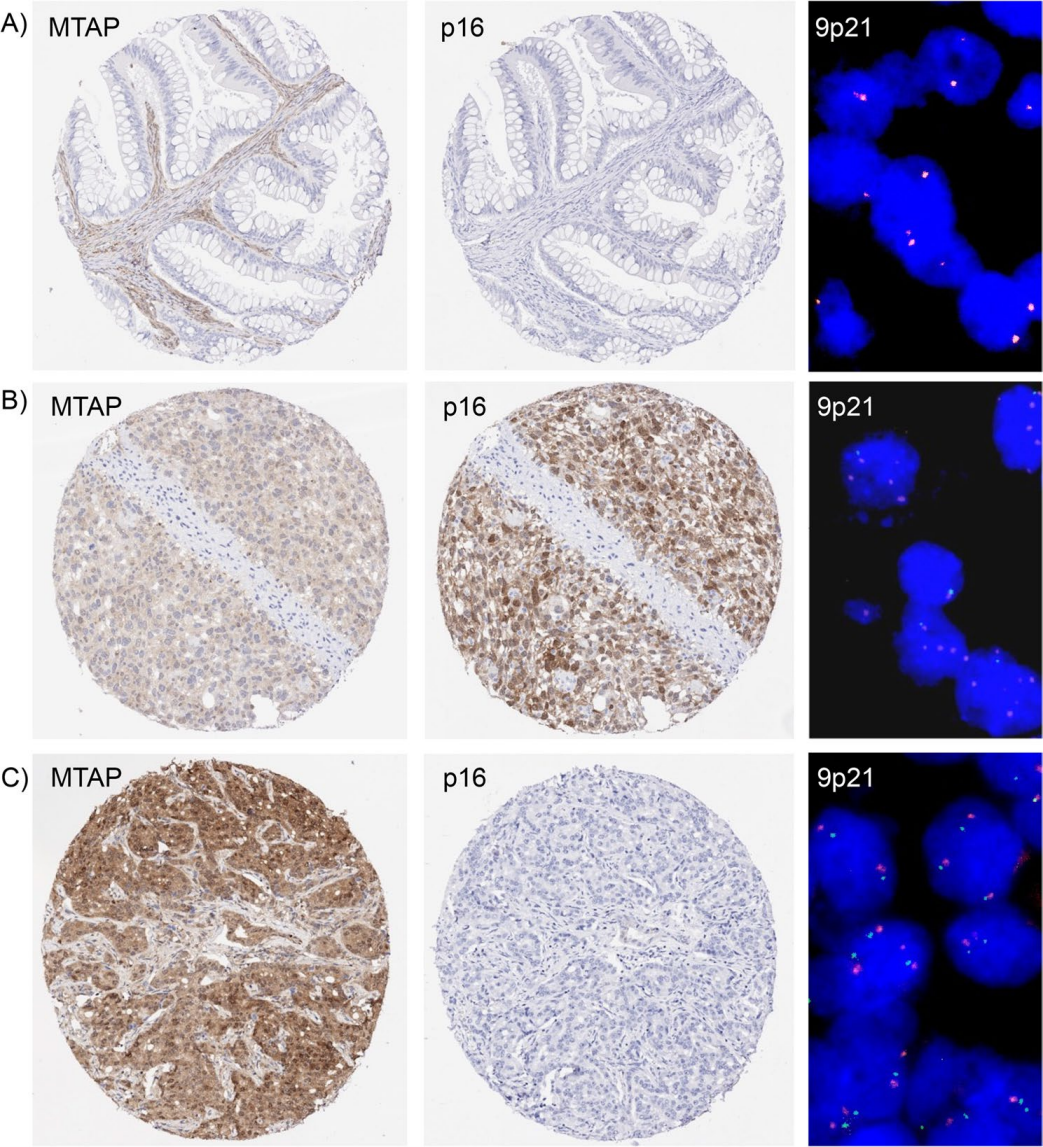
Examples of 9p21 copy number status and MTAP and p16 immunostaining. **A** mucinous ovarian carcinoma with 9p21 homozygous deletion and absence of MTAP and p16 immunostaining, **B** carcinosarcoma of the adnexa with heterozygous 9p21 deletion and weak MTAP but strong p16 immunostaining, and **C** prostatic adenocarcinoma with normal 9p21 copy number, strong MTAP staining but absence of p16 staining

**Fig. 5:**
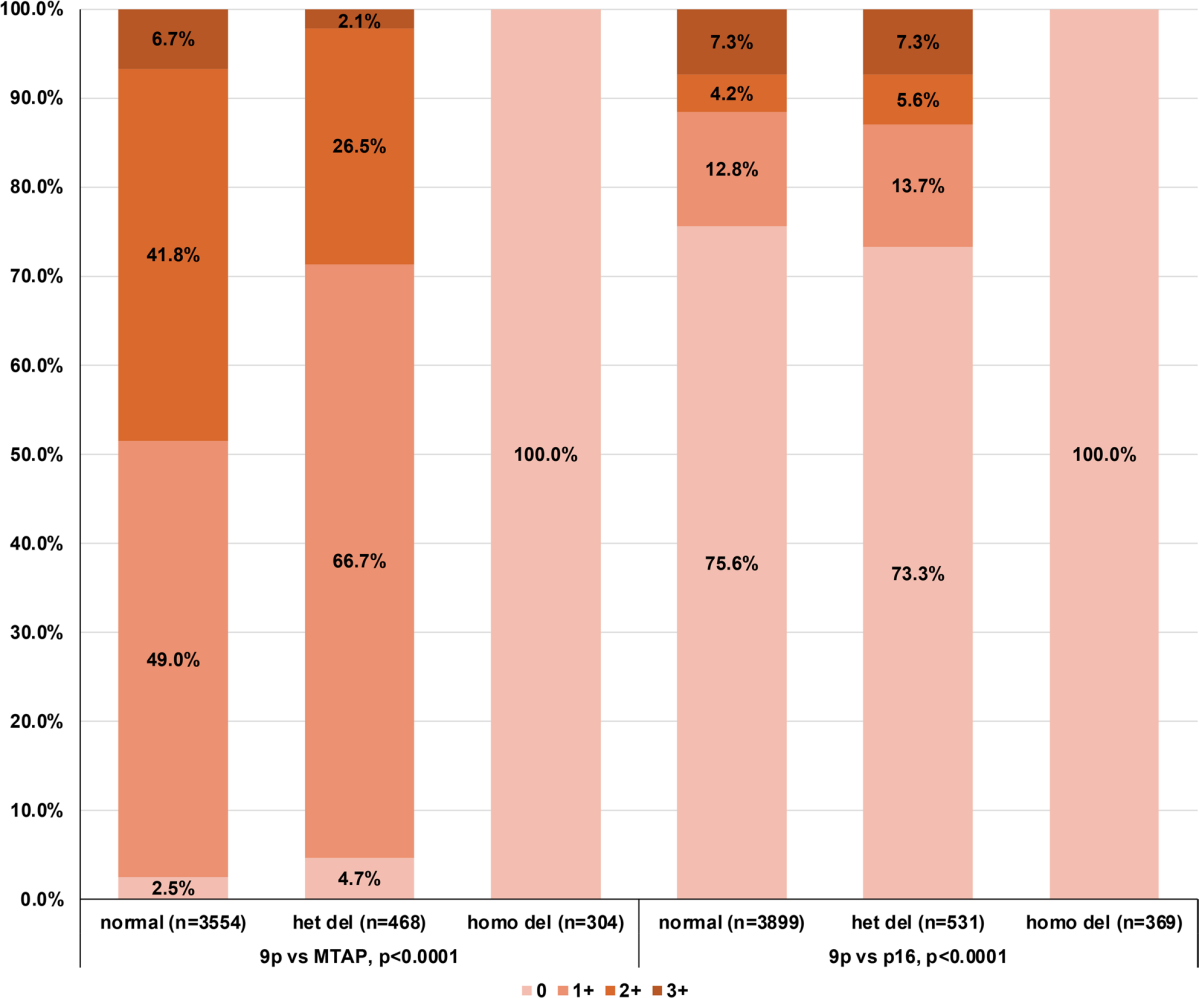
9p21 copy number status, MTAP and p16 immunostaining

Representative images of MTAP and p16 IHC are given in Fig. 4. The relationship between 9p21 deletion and a four-tier scoring of the intensity of immunostaining for MTAP and p16 are given in Fig. 5. Both proteins were not detectable in 304 cancers with homozygous 9p21 deletion and MTAP IHC results and 369 cancers with homozygous 9p21 deletion and p16 IHC results, but otherwise the results differed greatly between these proteins. The expression of MTAP was markedly lower in 468 heterozygously deleted cancers than in 3,554 wild type cancers (*p* < 0.0001). In contrast, the average staining intensity of p16 was slightly higher in 531 heterozygously deleted than in 3,899 wild type cancers although this difference did not reach statistical significance (*p* = 0.4564). In case of MTAP deficiency, 26.7% of 415 cases showed a normal (21.4%) or a heterozygous deleted (5.3%) 9p21 copy number status. In case of p16 negativity, 90.0% of 3,705 cases showed a normal (79.5%) or a heterozygous deleted (10.5%) 9p21 copy number status.

## Discussion

The successful analysis of 4,999 cancers from 125 different tumor entities revealed a heterozygous 9p21 deletion in 10.7% and a homozygous deletion in 7.4% of cancers. It is of note that FISH is the gold standard for detection of DNA copy number aberrations since DNA fragments can be visualized on a cell-by-cell basis and the analysis is not disturbed by the admixture of even large numbers of non-neoplastic cells. The validity of our FISH data for detection of homozygous deletions is also corroborated by a complete MTAP expression loss in 100% of our 304 homozygously 9p21 deleted tumors. Although our tumor cohort was artificially composed to include at least 50 cases for as many as possible tumor entities and therefore overrepresented less frequent cancer entities, our data might suggest that the rate of homozygously 9p21 deleted cancers is lower than the frequently referenced estimate of 10–15% 9p21 deletion (Beroukhim et al. 2010, Consortium 2020, Harrison et al. 2023).

That malignant mesothelioma, urothelial carcinomas, ductal adenocarcinoma of the pancreas, melanoma, and mucinous ovarian carcinomas were among the cancer entities with most frequent homozygous 9p21 deletions was to be expected based on our previous IHC data study evaluating 13,067 cancers from 149 different tumor types by MTAP IHC. Considering that concordance rates between MTAP IHC and 9p21 FISH ranged between 90.0% and 100% for the clinically relevant detection of homozygous deletions (Gorbokon et al. 2024, Gorbokon et al. 2025, Chapel et al. 2021, Lou et al. 2023, Sasaki et al. 2022, Kinoshita et al. 2018, Hida et al. 2017, Brich et al. 2020, Hamasaki et al. 2019, Chapel et al. 2020, Satomi et al. 2021), it can be assumed that IHC will become the gold standard method for the identification of MTAP deficient cancers. The even higher concordance rate of 100% in this study is most likely due to the use of consecutive tissue sections for MTAP IHC and 9p21 FISH. It is of note, however, that neuroendocrine tumors and many lymphomas show MTAP expression loss in the absence of 9p21 deletions (Gorbokon et al. 2024, Woollard et al. 2016, Hellerbrand et al. 2006). It is currently unclear, whether these tumors will respond similarly well to drugs targeting MTAP deficient tumors as cancers with homozygous deletions.

Considerable differences in the type of 9p21 deletions (homozygous vs. heterozygous) between tumor entities represent the key observation of this study. While homozygous deletions made up for 60–80% of 9p21 deletions in ductal pancreatic adenocarcinoma, urothelial carcinoma, malignant mesotheliomas, and mucinous ovarian carcinoma there were other tumor types such as cholangiocellular carcinomas, esophageal adenocarcinomas and liposarcomas where ≤ 30% of 9p21 deletions were homozygous while the fraction of homozygous deletions was ≤ 10% for other clinically important cancers such as endometrioid or high grade serous ovarian carcinomas. It is tempting to speculate that such marked differences may reflect cancer type specific roles and importants of involved 9p genes in different tumor entities and phenotypes. While cells from tumor types with frequent heterozygous but only rare homozygous 9p deletions may sufficiently benefit from a reduced function of one or several 9p genes, a complete inactivation of one or several genes may confer more significant benefits to cells from tumor entities where homozygous deletions predominate.

The targeting of several enzymes with augmented importance in MTAP deficient cells has resulted in striking clinical benefits in phase I and phase II trials including several reports of complete response in cancer patients who previously underwent several lines of chemotherapy (Engstrom et al. 2023, Herzberg and Johnson 2024, Brune et al. 2024, Bedard et al. 2024). The mechanisms leading to progressive disease in patients that initially responded to drugs targeting MTAP deficient cells have not been discovered. Molecular cancer heterogeneity is one possible cause for treatment escape. Considering that the development of MTAP deficiency by default requires two independent hits, each resulting in the loss of one gene copy, it can - for stochastic reasons - be assumed that the proportion of cancers with homozygous/heterozygous 9p21 deletions may be inversely linked to the expected proportion of cancers with heterogeneous 9p21 (MTAP) deficiency. The fraction of tumors with homogeneous MTAP deficiency should be highest in cancer types with more homozygous than heterozygous 9p21 deletions because homozygous deletions may have a stronger growth advantage in these entities than in those where heterozygous 9p21 deletions predominate. This assumption is in line with two of our previous MTAP studies showing that MTAP deficiency – mainly caused by homozygous 9p21 deletion - is highly homogeneous in carcinomas of the bladder (Gorbokon et al. 2025) and the pancreas (Gorbokon et al. 2025). In tumors that have MTAP deficiency only in subclones, the non-deficient clones will eventually outgrow the successfully treated deficient tumor population and result in cancer progression. If cancer entities with a low proportion of heterozygous 9p21 (MTAP) deletions do indeed had more frequent homozygous 9p21 (MTAP) deletions that are homogeneous in nature, the treatment of patients with urothelial carcinoma, mesothelioma or pancreatic adenocarcinoma might be less commonly infringed by cancer heterogeneity than in lung cancer patients. All these tumor entities are now commonly included into clinical trials with drugs targeting MTAP deficiency (Engstrom et al. 2023, Herzberg and Johnson 2024, Rodon 2024).

The evaluation of MTAP and p16 expression by IHC on consecutive TMA sections adjacent to those used for 9p21 deletion assessment enabled us to study the impact of these deletions on two important genes within the deleted area. The use of consecutive TMA sections assures optimally that identical tumor cell populations are analyzed for each parameter. It is of note that the level of standardization represents the key advantage of TMA studies which can hardly be achieved by other approaches. Studies comparing TMA results versus whole sections have demonstrated that the likelihood of a positive IHC staining increases markedly with the quantity of tissue analyzed (Li et al. 2017, Fernandez-Pol et al. 2023, Swierczynski et al. 2004). Authors have therefore suggested that TMAs should always contain multiple samples per tumor to increase representativity (Pedersen et al. 2014). We do not recommend following this recommendation because it increases the workload and the use of tissue by at least a factor of 2 without significantly increasing representativity for entire tumors. It must be recognized that even a whole section with a thickness of 2.5 μm containing a tumor area of 3 × 2 cm only represents 0.002% of the mass of a tumor measuring 5 cm in diameter. Even more important, the quantity of tumor tissue analyzed per patient is highly variable on whole sections (range: <1mm^2^ - >6cm^2^) while TMAs containing one sample of 0.6 mm tumor tissue per patient allow the analysis of standardized tumor mass per patient. TMAs thus always offer a standardized representativity of IHC data for entire tumor masses. Accordingly, the only study comparing whole sections and TMAs for their suitability to detect prognostic parameters have found superior results for TMAs in a cohort of > 550 breast cancers for one of three analyzed markers (Torhorst et al. 2001).

That MTAP staining was not seen in all 304 9p21 homozygous deleted cancers is in line with a reliable detection of *MTAP* deletions with our FISH probe. That a p16 staining was also not observed in any of these MTAP deficient cancers is in line with the concept that the *CDKN2A* (p16) gene is co-deleted in the vast majority of cases with *MTAP* deletion. Several previous studies had reported that *CDKN2A* deletions occur in 4–25% in the absence of *MTAP* deletions (Chapel et al. 2021, Zhang et al. 1996, Han et al. 2021) while *MTAP* deletions were hardly ever found without concurrent *CDKN2A* deletions (Chapel et al. 2021, Han et al. 2021). While it is expected that expression of a gene is completely lost in case of its homozygous deletion, our data show, that the impact of heterozygous deletions is less predictable. To better assess the impact of heterozygous 9p21 deletion on MTAP and p16 expression and to identify cases with p16 overexpression we used a four-tier classification (0, 1+, 2+, 3 + staining intensity) for the interpretation of the MTAP and p16 IHC staining. The markedly higher rate of reduced MTAP expression among heterozygously 9p21 deleted cancers than in wild type cancers suggests a gene dosage dependence of MTAP expression, which is well in line with data from us and others (Gorbokon et al. 2024, Gorbokon et al. 2025, Chapel et al. 2021). That p16 expression was even slightly higher in heterozygously deleted cancers than in wild type cancers demonstrates that p16 expression is not gene dosage dependent. That tumors with heterozygous 9p21 deletion failed to show a significant reduction of p16 expression and even demonstrated p16 upregulation in a fraction of cases is reflective of the involvement of p16 in important cancer related pathway alterations of which can cause compensatory upregulation such as inactivation of p53 or RB (Romagosa et al. 2011). In addition, that 90% of p16 negative cases do not show a homozygous 9p21 deletion is in line with our previous p16 study on 15,783 tumor samples of 124 tumor entities and 76 normal tissues showing that p16 expression levels in most normal tissues are below the detection level and that only a few cell types harbor detectable p16 immunostaining if standard antibodies and standard diagnostic IHC protocols are used. Therefore, in surgical pathology detectable p16 immunostaining is considered a feature of neoplastic disease / HPV infection in cervical epithelium (Kalof and Cooper 2006).

The main limitation of our study is the still low number of cases. By using the TMA approach, it would be possible to analyze 3 to 4 times more cases without substantial increase of the man power and reagents needed for tumor analysis. Higher number of cases would enable to obtained markedly stronger statistical data which is also desirable in the context of the high number of statistical analyses in the present study. It is another conceptional weakness of the FISH approach that exon deletion cannot be detected. Given the strong concordance of FISH data with IHC results this issue represents a minor problem. Inherently, the manual grading of staining intensity is semiquantitative. Various studies have, however demonstrated that – especially if a four-tier grading is used – that clinicopathological associations can be readily detected as little interobserver variability exists between the most relevant groups of 0 and 3 + cases.

In summary, the data from this study demonstrate that the proportions of heterozygous and homozygous 9p21 (*MTAP)* deletions vary markedly between tumor entities. The near perfect concordance between homozygous 9p21 deletions and absence of MTAP immunostaining further supports IHC as the gold standard method for detecting MTAP deficiency.

## Supplementary Information

Supplementary Material 1.

## Data Availability

All data generated or analyzed during this study are included in this published article.

## Abbreviations

FISH: Fluorescence in situ hybridization
IHC: Immunohistochemistry
MAT2A: methionine adenosyltransferase II alpha
MTAP: S-methyl-5′-thioadenosine phosphorylase
PRMT5: Protein arginine N-methyltransferase 5
SAM: S-adenosylmethionine
TMA: Tissue microarray
